# Editorial: Exploring causal risk factors for metabolic and endocrine disorders

**DOI:** 10.3389/fendo.2024.1357999

**Published:** 2024-02-01

**Authors:** Di Liu, Haifeng Hou, Xiao Wang, Youxin Wang

**Affiliations:** ^1^ Center for Biomedical Information Technology, Shenzhen Institutes of Advanced Technology, Chinese Academy of Sciences, Shenzhen, Guangdong, China; ^2^ School of Public Health, Shandong First Medical University & Shandong Academy of Medical Sciences, Jinan, China; ^3^ Department of Clinical Sciences, Center for Primary Health Care Research, Lund University/Region Skåne, Malmö, Sweden; ^4^ School of Public Health, North China University of Science and Technology, Tangshan, China

**Keywords:** metabolic and endocrine disorders, risk factor, causal association, mendelian randomization, meta-analysis

Metabolic and endocrine disorders remain a significant global health concern, with their prevalence steadily increasing in recent years. These disorders, including conditions but not limited to hypertension, diabetes and its complications, dyslipidemia, obesity, metabolic syndrome, hyperuricemia, non-alcoholic fatty liver disease, polycystic ovary syndrome, thyroid disorders, parathyroid disorders, pituitary disorders, and adrenal disorders, not only cause of morbidity and mortality and affect well-being but also pose substantial challenges to healthcare systems worldwide (1). The rising burden of metabolic and endocrine disorders has prompted extensive research to understand their causal risk factors. Identifying these factors is crucial for developing effective preventive strategies and improving public health outcomes. The main purpose of the editorial is to summarize the current state of research on causal risk factors for metabolic and endocrine disorders, highlighting the need for further investigation and collaboration in this critical area of study.

Etiological inference is the core of epidemiological research, which informs etiological modeling and prevention efforts. Large-scale epidemiological studies, genetic analyses, and clinical trials are the main study design for etiological inference, which have specific advantages and disadvantages that can complement each other to some extent (2, 3). In recent years, with the large-scale release of GWAS data, Mendelian randomization study has been widely applied in causal inference (4, 5), in which genetic data may partially address the limitations of confounding and reverse causality and provide more convincing evidence to explain the underlying causal associations. It remains challenges associated with identifying causal factors due to complex interactions between genetic, environmental, and lifestyle factors.

In this Research Topic, cross-sectional, cohort, meta-analysis, and Mendelian randomization study designs were used to explore the risk factors for metabolic and endocrine disorders. As metabolic and endocrine disorders could act as risk factors for diseases or adverse outcomes of risk factors, several studies investigated the bidirectional causal relationship of metabolic and endocrine disorders as exposures or outcomes. In addition, there is a mutual relationship between metabolic disorders and endocrine dysregulation. When there is dysfunction in the endocrine system, it can lead to metabolic abnormalities and the development of metabolic disorders. On the other hand, metabolic disorders can also affect the functioning of the endocrine system.

The findings of Tan et al., determine the genetic structure shared between NAFLD and T2D, offering a new reference for the genetic pathogenesis and mechanism of NAFLD and T2D comorbidities.

Chen et al.‘s systematic review and meta-analysis indicates that clopidogrel might be a modifiable and causal risk factor of hypoglycemia, especially in the Asian population.

Cheng et al. demonstrate that levels of triglycerides and remnant-C, but not TC or LDL-C, were associated with NAFLD outcomes independent of other risk factors, in the middle aged and elderly subset of the Chinese population, especially those who were women, non-CVD status, non-diabetes status and middle BMI status (24 to 28 kg/m^2^).

In a Mendelian randomization study, Du et al. discover an important role of polycystic ovary syndrome in the development of chronic kidney disease, underlying the importance of regular follow-up of renal function in patients with polycystic ovary syndrome.

Xu et al. show that genetically predicted causal relationship of inflammatory bowel disease with bone mineral density and osteoporosis using evidence from two-sample Mendelian randomization.

Yang et al. demonstrate the potential role of bile acids in bone metabolism among T2DM patients in a north China population.

Using an ethnic-specific GWAS of 146 metabolites and 1-sample Mendelian randomization analyses in a UK multi-ethnic birth cohort, Fuller et al. confirm and demonstrate the presence of ethnic-specific causal relationships between metabolites and dysglycemia in mid-pregnancy in a UK population of SA and WE pregnant women.

Yang et al. assess the usefulness of a newly proposed metabolic score for visceral fat in predicting future diabetes, and finds that Metabolic Score for Visceral Fat is positively correlated with diabetes risk.

Ren et al. show that increased serum Cat-S is associated with the progression of albuminuria and decreased renal function in T2DM patients.

Nasr et al. conclude that serum estradiol levels may have a causal effect on kidney function, based on evidence from a MR analysis.

Gatta et al. show that 50% of patients developed autoimmune polyglandular syndrome type 4 within the first ten years, don’t suggest any particular follow-up time and don’t specify any particular disease.

Li et al. find that psychological stress is associated with hypertension both in cross-sectional and MR studies, suggesting targeting hypertension-related factors in interventions might improve mental and metabolic health.

Zhang et al. explore the risk factors for cognitive impairment (CI) in patients with type 2 diabetes mellitus (T2DM), screen potential therapeutic drugs for T2DM-CI, and provide evidence for preventing and treating T2DM-CI, using artificial intelligence interpretation and graph neural networks.

Based on data from the national health and nutrition examination survey (2007–2018), Tan et al. find an association between serum uric acid and hypertriglyceridemia.

Using data from the NHANES 2007-2014, Liu et al. show an association of serum 25-hydroxyvitamin D with urinary incontinence in elderly men.

Zhang et al. use clinical and genetic data from different public biological databases and perform two-sample and two-step Mendelian randomization analyses. The study suggests that exposure to heavy air pollutants causally increases risks for obesity.

Ren et al. investigate distinct risk factors for hyperuricemia in native Tibetan and immigrant Han populations in Tibet, China, and confirms the distinctive biochemistry between Tibetans and Hans.

Si et al. indicate a causality between polycystic ovary syndrome and susceptibility and severity of COVID-19 using a bidirectional Mendelian randomization study.

This unique insight will significantly contribute to the development of innovative prevention strategies for metabolic and endocrine disorders. This Research Topic aims to bring together important research findings from distinguished researchers and scientists worldwide. We strive to provide readers with ample opportunities to stay informed about the latest research and cutting-edge advances in this field through this collaboration. By fostering interdisciplinary collaboration and facilitating knowledge sharing, we firmly believe that we can pave the way for new avenues of research in the prevention of metabolic and endocrine disorders.

## Author contributions

YW: Conceptualization, Funding acquisition, Supervision, Writing – original draft, Writing – review & editing. DL: Writing – original draft, Writing – review & editing. HH: Writing – original draft, Writing – review & editing. XW: Writing – original draft, Writing – review & editing.
